# Hydroquinone Ecotoxicity: Unveiling Risks in Soil and River Ecosystems with Insights into Microbial Resilience

**DOI:** 10.3390/toxics12020115

**Published:** 2024-01-29

**Authors:** Antonio Valenzuela, Diego Ballestero, Cristina Gan, Guillermo Lorca, Elisa Langa, María Rosa Pino-Otín

**Affiliations:** Faculty of Health Sciences, Universidad San Jorge, Villanueva de Gállego, 50830 Zaragoza, Spain; avalenzuela@usj.es (A.V.); dballestero@usj.es (D.B.); cgan@usj.es (C.G.); glorca@usj.es (G.L.); elanga@usj.es (E.L.)

**Keywords:** hydroquinone, acute toxicity, *Daphnia magna*, *Aliivibrio fischeri*, *Allium cepa*, *Eisenia fetida*, microbial communities

## Abstract

Despite widespread industrial use, the environmental safety of hydroquinone (HQ), a benzene compound from plants used in processes like cosmetics, remains uncertain. This study evaluated the ecotoxicological impact of HQ on soil and river environments, utilizing non-target indicator organisms from diverse trophic levels: *Daphnia magna*, *Aliivibrio fischeri*, *Allium cepa*, and *Eisenia fetida*. For a more environmentally realistic assessment, microbial communities from a river and untreated soil underwent 16S rRNA gene sequencing, with growth and changes in community-level physiological profiling assessed using Biolog EcoPlate™ assays. The water indicator *D. magna* exhibited the highest sensitivity to HQ (EC_50_ = 0.142 µg/mL), followed by *A. fischeri* (EC_50_ = 1.446 µg/mL), and *A. cepa* (LC_50_ = 7.631 µg/mL), while *E. fetida* showed the highest resistance (EC_50_ = 234 mg/Kg). Remarkably, microbial communities mitigated HQ impact in both aquatic and terrestrial environments. River microorganisms displayed minimal inhibition, except for a significant reduction in polymer metabolism at the highest concentration (100 µg/mL). Soil communities demonstrated resilience up to 100 µg/mL, beyond which there was a significant decrease in population growth and the capacity to metabolize carbohydrates and polymers. Despite microbial mitigation, HQ remains highly toxic to various trophic levels, emphasizing the necessity for environmental regulations.

## 1. Introduction

Hydroquinone (HQ) is an aromatic compound found in various forms as a natural product from plants and animals [1]. It also has widespread applications in human and industrial activities, likely serving as the major benzene metabolite.

HQ serves various applications as a reducing agent in photographic developers, an antioxidant, and a polymerization inhibitor in the production of monomers, polymers, dyes, pigments, rubber products, and various chemicals. Historically, it has been used in the cosmetics industry for applications such as skin lightening, hyperpigmentation treatment, anti-aging products, sunscreen formulations, coating fingernails, and hair dyes.

However, according to the harmonized classification and labelling (ATP01) ap-proved by the European Union (European Chemical Agency, ECHA) [2], this substance can cause serious health damage, including genetic defects, and its cosmetic use is prohibited by Regulation (EC) No. 1223/2009, with exceptions such as professional uses (hair dyeing and artificial nail systems). Since the 1960s, it has also been used as a medical product in topical treatments for acne scars, post-inflammatory hyperpigmentation, and certain types of dermatitis, and this use continues to be authorized by the European Medicines Agency (EMA) and the Food and Drug Administration (FDA) [3] in pharmaceutical and over-the-counter products (Medical Pharmaceutical Formulary, PharmaBooks, 2010).

While there is a minor natural release of HQ by plants and animals, industrial uses and their discharges are the main cause of the dispersion of HQ into the environment [4,5,6].

Although numerous techniques have been developed for its detection, there are not many studies that reveal concentrations at which this product can be found in the environment. HQ has been detected in bleachery effluents from kraft pulp production [4] at concentrations up to 40 µg/L and as an intermediate metabolite in many other effluents such as phenolic resins [5] and organophosphate esters [6]. Moreover, HQ is the key intermediate of many degradation pathways, such as paracetamol [7,8], bisphenol A [9], or the disinfectant chlorophene [10] among many others. In acetaminophen-contaminated sludge from wastewater purification plants, HQ is one of the most commonly detected intermediates [11]. Additionally, the formation of HQ and other derivatives in the early stages of phenol oxidation appears to increase the toxicity of phenolic wastewaters, making HQ more toxic than the initial product [12,13].

Wastewater containing this product ends up in watercourses. Thus HQ has been detected in river water at different sampling sites and in different months [14,15] at concentrations up to 1000 µg/L and even at similar concentrations in tap water [16,17]. It has also been detected in stream water near public landfills [18].

In addition to these pathways, other routes can carry this product to the soil. For example, HQ has been shown to be a byproduct of the degradation of the pesticide pentachlorophenol, dispersed globally in soils [19] as well as he commonly used herbicide fenoxaprop-p-ethyl [20]. HQ is also applied to soil as a urease inhibitor [21].

The classification provided by companies to ECHA in the European Union regulation Registration REACH [22] (Authorization, and Restriction of Chemicals) identifies this substance as highly toxic to aquatic life with long-lasting effects.

Some studies show high HQ toxicity to aquatic organisms, including algae (EC_50_: 50 to 11,000 µg/L) [23], with cyanobacterial species (such as *Microcystis aeruginosa*) being much more susceptible than coccal green algal species. Green musk chara exhibited phytotoxicity at 1.1 µg/L [24]. HQ is also toxic to mollusks [25] and adverse effects are documented in fish species, e.g., zebrafish embryos (EC_50_: 3.2 mg/L to < 0.1 mg/L) [26], rainbow trout, and fathead minnows [27,28].

Little is known about the effect of HQ on terrestrial invertebrates. It appears to be toxic to snails [29] and insects such as *Apis mellifera* [30]. Phytotoxic effects have also been reported on plants of the genus *Vallisneria* and *Lemna*. HQ was lethal to rice above 5 mM [24] and acts by inhibiting germination of *Cucuvis sativus* seeds (103.9 mg/L of HQ inhibits 50% of seeds germination) [31].

Few studies have examined HQ’s impact on entire environmental communities. Some focus on its effects on microbial communities in wastewater treatment plants [32,33,34,35], however little is known about its ecotoxicity in river or edaphic microbial communities.

HQ has been proposed as a urease inhibitor in agricultural soils to minimize ammonia volatilization, enhancing nitrogen utilization efficiency for plant benefit [21]. This would be another route of entry of HQ into soil systems. Limited evidence suggests minor impacts on soil microorganism community structure when used alone or in combination with other urease inhibitors [36,37].

Therefore, despite the apparent toxicity of this product and its abundant dispersion in the environment, there are still gaps in the interpretation of the HQ impact on the environment through individual non-target indicators. Moreover, studies that include communities of organisms such as bacteria and have a more realistic environmental point of view are lacking. To maintain a healthy ecosystem, it is crucial to adopt an integrated approach that considers not only individual species but also their interconnected relationships, as multiple species coexist closely within it.

Therefore, the objectives of this study are:

(a) To evaluate the toxicity of HQ on key indicator organisms in soil and water for which little information exists.
(b) To evaluate for the first time the toxicity of HQ on 16 S rRNA gene-sequenced fluvial and soil microbial communities in order to more realistically assess the impact on these environments.

Thus, by studying the impact of HQ on different trophic levels, individuals, and communities, we can obtain a complete perspective of the impact of this compound on the environment.

## 2. Materials and Methods

### 2.1. Reagents

HQ (CAS: 123-31-9) was obtained from Acofarma (Barcelona, Spain) with a purity of 99.5%. Table 1 shows the main properties of HQ.

### 2.2. Daphnia Magna Assay

The impact of HQ on *D. magna* was investigated following the standard procedure outlined in Daphtoxkit FTM magna (1996), reference DM121219 from Vidrafoc (Barcelona, Spain), and in accordance with OECD 202 (2004) [41] guidelines. The kit was stored in darkness at 5 °C until use. Initially, *D. magna* eggs were incubated at 22 °C under 6000 lx light conditions using a TOXKIT model CH-0120D-AC/DC incubator (ECOTEST, Valencia, Spain) for 72 h. Subsequently, neonates were fed with one vial of spirulina microalgae for 2 h and exposed to solutions of HQ at concentrations of 0.01, 0.1, 0.5, 1, and 2 µg/mL, dissolved in synthetic freshwater (ISO 6341, 2012) [42], for 24 h in the same incubator but under complete darkness.

The pH was maintained between 7–7.5, rendering adjustments unnecessary. Each concentration was evaluated using 5 replicates, each containing 5 organisms, with synthetic freshwater serving as the negative control. After 24 h of exposure, daphnias showing no movement for 15 s after gentle agitation were considered immobile. The obtained results were utilized to calculate the LC_50_, representing the concentration of the compound resulting in 50% lethality.

### 2.3. Aliivibrio Fischeri Assay

The assessment of *A. fischeri* acute toxicity was carried out by evaluating bioluminescence inhibition caused by the presence of HQ, in accordance with the established protocol outlined in (ISO 11348-3, 2007) [43]. The strain utilized for this analysis was *A. fischeri* NRRL-B-11,177, obtained from Macherey-Nagel (ref. 945 006, Dueren, Germany). Lyophilized *A. fischeri* were reconstituted using the provided reactivation solution and stored at 4 °C for 5 min.

HQ stock solutions were prepared using a 2% NaCl stock solution at various concentrations: 0.1, 1, 10, 100, and 1000 µg/mL. Solutions did not require pH adjustment. The assay was conducted in quadruplicate, in four tubes containing bacteria and each HQ concentration solution, and one tube with just a 2% NaCl stock solution serving as the negative control.

To initiate the assay, baseline luminescence was measured. Subsequently, 0.5 mL of each HQ dilution prepared for testing was added to the corresponding tubes. Within the recommended time frame specified by the standard, after a 30-min incubation period, the second measurement of luminescence inhibition was conducted. Measurements were recorded using a Biofix^®^ Lumi-10 luminometer (Macherey-Nagel, Dueren, Germany). The test endpoint was determined by the reduction in bacterial light production. The EC_50_ values were expressed as a percentage of luminescence inhibition and calculated in comparison to the control.

### 2.4. Allium cepa Assay

Bulbs from the *A. cepa* species, specifically the Stuttgarter Riesen variety with a 14/21 gauge, were obtained from the Fitoagrícola Company (Castellón, Spain). In the preparatory phase of the experimental setup, the young bulbs underwent a peeling process to ensure the preservation of root ring integrity. Acute toxicity experiments involving *A. cepa* were conducted following the methodology outlined by Fiskesjö [44].

The bulbs were carefully arranged in 15 mL tubes, and mineral water (Aguas de San Martín de Veri S.A., Huesca, Spain) was selected as the growth medium due to its suitable calcium and magnesium content, as detailed on the product’s official website (https://www.veri.es/es/el-producto, accessed on 10 April 2023). Ecotoxicological tests were conducted with 12 replicates for each concentration: 0.03, 0.3, 3.0, 30, and 300 mg/L. The negative control consisted of water alone. The bulbs were cultivated in an incubator under light conditions at a temperature of 25 °C for a duration of 72 h, with the test solutions being refreshed every 24 h. The endpoint for assessment was the measurement of root growth inhibition, and the EC_50_ was calculated as part of the analysis.

### 2.5. Eisenia Fetida Assay

Mature individuals of *E. fetida* were obtained from composters located at Todo Verde (Madrid, Spain). Prior to the commencement of the tests, the earthworms underwent a 15-day acclimatization period in a substrate conditioned with sphagnum peat provided by the Spanish Flowers Company (Barcelona, Spain). The earthworms were carefully maintained under stable environmental conditions, specifically at a temperature range of 18–25 °C, pH levels between 7.5–8, and humidity levels maintained at 80–85%.

For the ecotoxicity assessment, adult earthworms aged above 60 days, with clitellum, and weighing between 300–600 mg, were selectively chosen for the experiments. The toxicity tests adhered to the guidelines outlined in OECD 207 (1984) [45] methodology, as previously detailed in research [46]. These tests were conducted in a standardized soil substrate comprising quarzitic sand and kaolinic clay (both from Imerys Ceramics España, S.A., Castellón, Spain), and sphagnum peat (Verdecora vivarium, Zaragoza, Spain) in a proportionate ratio of 7:2:1.

Polypropylene containers, equipped with perforated lids to facilitate ventilation and minimize moisture loss, were used for the experiments. Each container was filled with 600 mg of the artificial soil mixture. Within each container, ten earthworms were placed alongside HQ solutions, with final concentrations set at 0.1, 1.0, 10, 100, and 1000 mg/Kg. The moisture content of the substrate was adjusted to 35–45% of the dry soil weight using deionized water. Negative controls were established following the same procedural steps but without the inclusion of HQ. Each concentration level was subjected to triplicate testing.

Throughout the experimental period, the containers were carefully maintained under controlled environmental conditions, specifically at a temperature of 20 ± 2 °C, relative humidity ranging between 80–85%, and light intensity maintained at 400–800 lx. The assessment of earthworm mortality was conducted 14 days after the initiation of treatment, and subsequently, the LC50 values were calculated.

### 2.6. River and Soil Microorganisms Community Assay

#### 2.6.1. River Samples

In October 2022, water samples were collected from the Gallego River (Zaragoza, Spain) for genetic and chemical analyses, along with Biolog EcoPlates™ assays (Tiselab S.L., Barcelona, Spain) and transported to the laboratory following ISO 19458:2006 [47] procedures by AENOR. In situ measurements revealed a water temperature of 17 °C using a Nahita thermometer (ICT S.L., La Rioja, Spain), pH 7.5 determined with PanReac AppliChem A011435 (Darmstadt, Germany), and a conductivity of 2.8 mS measured with a Hanna HI8733 (Merck Madrid, Spain) conductivity meter. Analysis of the river water can be seen in Table A1.

For genetic analysis, microorganisms were obtained from 5 L of river water, filtered through a 0.22 μm cellulose nitrate filter Sartorius (Göttingen, Germany) using a vacuum flask. The filtered microorganisms were reconstituted in a sterile Falcon tube with 50 mL of phosphate-buffered saline (PBS), subjected to centrifugation at 5000× *g* for 10 min, and the resulting pellet preserved at −80 °C for subsequent sequencing.

To prepare for ecotoxicity assays, 1 L of river water underwent filtration through a 70 μm nylon sieve (Becton Dickinson, Madrid, Spain) to remove debris. The filtered water was stored at 4 °C in the dark until used in Biolog EcoPlates™ experiments. Additionally, two liters of the same water were promptly transported to Laboratorios Valero Analítica (Zaragoza, Spain) on the sampling day for physicochemical analysis (Table A1).

#### 2.6.2. Soil Samples

In November 2022, soil samples were obtained from a pesticide-free crop field at the Agri-food Research and Technology Center of Aragon (CITA, Zaragoza, Spain). The soil analysis was conducted by the CITA Soil and Irrigation Unit, and detailed results are available in Table A2.

For genetic analysis, 20 g of soil was mixed with 100 mL of sterile water. After 30 min of stirring under sterile conditions and a settling period of 1 h, 10 mL of the sample underwent sonication for 1 min, followed by centrifugation at 1000× *g* for 10 min. In a sterile environment, the supernatant was collected, and soil microorganisms were isolated using a 0.22 μm cellulose nitrate filter (Sartorius Spain SA, Madrid, Spain) and a vacuum flask. The filter content was washed with sterilized PBS, followed by centrifugation at 5000× *g* for 10 min. The resulting pellets were collected and stored at −80 °C for subsequent sequencing.

Before ecotoxicity assays, 10 g of soil underwent preliminary sieving using a 2 mm sieve (Becton Dickinson, Madrid, Spain). The pre-sieved soil was mixed with 95 mL of sterile water in an Erlenmeyer flask for 30 min, followed by a settling period of 1 h. After settling, 10 mL of the upper portion of the flask was transferred to Falcon tubes, experiencing centrifugation at 1000× *g* for 10 min, with the sterile collection of the supernatant. This process was repeated five times, and the cumulative supernatant was passed through a 70 μm nylon sieve (Becton Dickinson, Madrid, Spain) to remove suspended soil debris, ensuring a purified sample suitable for inoculation in Biolog plates.

#### 2.6.3. Genetic Sequencing of River and Soil Microorganisms

The preprocessed solution from the conclusion of Section 2.6.1 and Section 2.6.2 underwent an additional filtration step utilizing Sartori 0.2 µm cellulose nitrate filters that had been thoroughly rinsed with a PBS (Phosphate Buffered Saline) solution with a pH of 7.5. The PBS solution was collected in Falcon tubes and centrifuged at 5000× *g* for 10 min. Following careful removal of the supernatants, the resulting pellets were frozen at −80 °C for subsequent genetic analysis using the Froilabo, Trust −80 °C system.

DNA extraction was performed employing the AllPrep^®^ PowerViral^®^ DNA/RNA Kit (QiaGen, Barcelona, Spain), following the manufacturer’s guidelines. Subsequently, the purified DNA samples were quantified fluorimetrically using Picogreen^®^, and 1.5 ng of input DNA from each sample was employed to amplify the V3-V4 region of the 16S rRNA gene. The V3-V4 specific PCR consisted of 21 cycles and was performed using Q5^®^ Hot Start High-Fidelity DNA Polymerase (New England Biolabs, Ipswich, MA, USA) and 100 nM primers. After amplification, positive 16S-derived bands were assessed through agarose gel electrophoresis, and DNA products were diluted. A second PCR, consisting of 13 cycles, was carried out in the presence of 400 nM primers, belonging to the Access Array Barcode Library for Illumina Sequencers (Fluidigm, CA, USA) collection. This second PCR finalizes the Illumina library construction and assigns each sample a unique barcode. Following individual library preparation, samples were assessed for size and concentration using a Tape Station (Agilent, Madrid, Spain), and an equimolar pool was created. The pool was purified using AMPure beads and quantified via quantitative PCR using the “Kapa-SYBR FAST qPCR kit for LightCycler480” (Sigma-Aldrich, Madrid, Spain) and a reference standard for quantification.

The pool of amplicons was denatured before being loaded onto a flowcell at a concentration of 10 pM, where clusters were formed and subjected to sequencing using a “MiSeq Reagent Kit v3” in a 2 × 300 pair-end sequencing run on a MiSeq sequencer.

The resulting fastq files were generated using the bcl2fastq tool integrated into the Illumina sequence workflow. Phylogenetic analysis was conducted using the 16S Metagenomics app of Base Space v1.1.0 (Illumina, Madrid, Spain), with Greengenes (13_5) serving as the database for taxonomic assignment.

#### 2.6.4. Community-Level Physiological Profiling (CLPP) of River and Soil Microorganisms

To investigate the impact of HQ on the metabolic activity of microbial communities in water and soil, Biolog EcoPlate tests from Tiselab S.L. (Barcelona, Spain) were utilized. This method allowed monitoring changes in the utilization of 31 diverse carbon sources, as detailed in previous studies [48]. For ecotoxicity assessment, solutions containing HQ at varying concentrations (0.1, 10, and 100 µg/mL) were prepared in sterile water, each with a final volume of 150 μL, and added to the wells of a Biolog plate under sterile conditions.

Prefiltered river water (see Section 2.6.1) or the supernatant obtained from the soil sample (Section 2.6.2) was used for studying the metabolic activity of river and soil microorganisms, respectively. The pH of these solutions was maintained between 6 and 7. Each concentration was tested in triplicate, with all procedures conducted under sterile conditions within a flow chamber. After preparation, the plates were placed in the dark at a temperature of 25 °C for 7 days, ensuring sterile conditions throughout the experiment.

Optical density (OD) measurements at a wavelength of 590 nm were taken immediately after inoculation and then once daily. A Synergy H1 Microplate reader (BIO-TEK, Dallas, TX, USA) with Gen5™ (version 2.0) data analysis software was used for this purpose. The carbon utilization rate was determined by assessing the reduction of tetrazolium violet redox, following the method outlined by Pohland [49].

### 2.7. Statistics and Graphic Representation

To establish dose–response curves for *D. magna* mobility, *E. fetida* survival, *A. cepa* root elongation, and *A. fischeri* luminescence, logit logistic regression was applied using XLSTAT software (version 2014.5.03, Addinsoft 2024). This approach facilitated the calculation of LC_50_ and EC_50_ values, along with their corresponding standard errors (SE). The statistical significance of the dose–response models was assessed through a chi-squared test.

Microbial activity for each Biolog EcoPlate was quantified using Average Well Color Development (AWCD), following the methodology outlined by Garland and Mills [50], as cited in previous studies [51].

Graphical representations of the results were generated using appropriate visualization techniques, and Equation (1) was employed:

(1) $$AWCD=\sum_{i=0}^{i=7}\left({OD}_{t=xi}-{OD}_{t=x0}\right)\;$$

*OD_i_* represents the optical density value from each well at any given time after subtracting the *OD_t_* _=_ *_X_*_0_ from the *OD_t_* _=_ *_Xi_* of that well.

The relationship between AWCD values from the three replicates and the significance of differences were assessed using a Student’s *t*-test for two independent samples, performed with XLSTAT software (version 2014.5.03). The coefficient of variation (CV) is used to assess the relative dispersion of absorbance data in the three replicates.

Finally, AWCD curves were fitted to a logistic model (Equation (2)) for sigmoid microbial growth, as described in previous studies [52] using the Excel Solver (Microsoft 365) complement:

(2) $$AWCD=\frac{Cmax}{1+e^{b-rt}}\;$$

Here, *Cmax* represents the carrying capacity or the maximum achievable population density, *r* is the intrinsic rate of population increase, and *b* is a fitting parameter.

## 3. Results and Discussion

### 3.1. Impact of Hydroquinone on Daphnia magna

**Figure 1:**
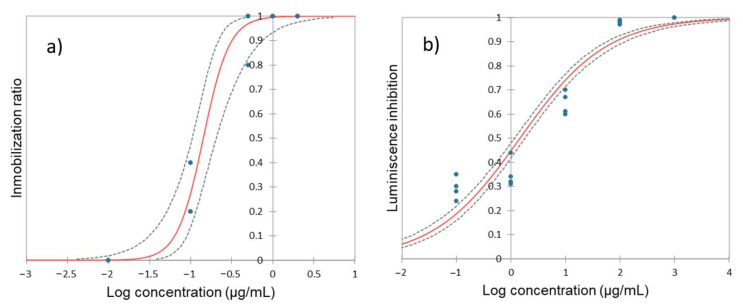
Dose–response curve for (**a**) *Daphnia magna* and for (**b**) *Aliivibrio fischeri* after 24 h and 30 min of exposure to hydroquinone, respectively. Red line represents the model and dashed lines indicate the confidence limits (95%). The points indicate the values of the replicates, in some cases the values may overlap.

Figure 1a shows the *D. magna* dose–response curve to HQ.

The calculated EC_50 24 h_ for HQ is 0.142 (0.104–0.204) µg/mL, indicating high toxicity of this product on *D. magna*. The toxic effects of HQ on this organism have been documented in previous studies [53] and when calculating the EC_50_, the values obtained after 24 h of exposure, are very similar to ours, with EC_50_ = 0.150 µg/mL [54,55]. After 48 h of exposure, values are slightly higher at 0.25–0.28 µg/mL [20]. Interestingly, another aquatic crustacean species, also belonging to Branchiopoda, *Ceriodaphnia dubia*, shows a very similar sensitivity to HQ as *D. magna* with EC_50_ values of 0.15 µg/mL as well [27].

*D. magna* is a good indicator of water quality since it is exposed to toxics through a dual pathway: surface exposure and also through its diet as it is a filter-feeding organism. HQ is a relatively small molecule (MW = 110.11 g/mol) and electrically neutral, with a pKa of approximately 9.9 and 11.6 [40], which might facilitate its passage through cell membranes. Changes in membrane permeability can affect the integrity of the cell membranes of *D. magna*, subsequently altering cellular homeostasis and leading to cell death. However, it is not a very lipophilic compound (LogKow = 0.59) [40].

On the other hand, it is soluble in water (73 g/L at 25 °C) [38] which enhances its bioavailability. Therefore, the digestive tract may be the main route of exposure to these organisms, facilitating the entry of HQ into *D. magna*, which could lead to cardiac [56] and nervous [57] disturbances. It could also act by inducing oxidative stress [58] or affecting the protein content in the hemolymph, as observed in other invertebrates [59]. Similar to benzene, HQ can inhibit the activity of certain enzymes such as topoisomerase II [60], negatively impacting essential cellular processes for the survival of *D. magna*. This, in conjunction, would explain the high toxicity of HQ observed on this organism.

### 3.2. Impact of Hydroquinone on A. fisheri

The toxicity of HQ to the bacteria *A. fischeri* is illustrated in Figure 1b and the obtained EC_50_ was 1.446 (1.155–1.796) µg/mL. Limited data exist on the toxicity of HQ to *A. fischeri*, as studies typically focus on the toxicity of byproducts, including HQ, generated during the decomposition of various products such as paracetamol [61], benzidine [62], benzoquinone [63], sulfamethoxazole [64], sulfanilamide [65] or clofibric acid [66] among others. It is noteworthy that almost all studies agree that HQ is one of the most toxic byproducts, even more than the original product.

The EC_50_ value for *A. fischeri* exposed to HQ (as dimethomorph intermediate on TiO_2_ suspension) in a 2% NaCl solution was measured at 0.08 mg/L [67] but the exposure time was only 5 min. A. Santos et al. [68] reported an EC_50_ of 0.041 mg/L (15 min) in *A. fischeri* during the catalytic oxidation of phenol. These results are challenging to compare due to different experimental conditions, and in our case, the exposure was for 30 min. Nevertheless, all results suggest that HQ is highly toxic to this aquatic indicator.

The Gram-negative outer covering of *A. fischeri* may partially shield the bacterium from intracellular exposure to HQ, acting as a selective barrier. Due to its size, HQ may face challenges in traversing the porins of the outer membrane of the Gram-negative wall or interacting with its lipopolysaccharides. Alternatively, it could be expelled by efflux pumps. This may explain its somewhat lower toxicity compared to *D. magna*. However, once inside the prokaryotic cell, it is likely to have toxicity mechanisms similar to those observed in *D. magna*.

To the best of our knowledge, no information is available regarding the mechanism of action of HQ on *A. fisheri.* However, documented inhibitory effects on the growth of pathogenic bacteria such as *Pseudomona aeruginosa*, *Klebsiella pneumoniae*, and *Escherichia coli* have been reported [69,70,71]. Additionally, studies on other bacteria within the genus *Aliivibrio* [72,73] observed antimicrobial activity of HQ derivatives. Interestingly, these derivatives appear to downregulate genes of *Aliivibrio* spp. implicated in motility, protease synthesis, indol, and capsular polysaccharide production, suggesting a potential mechanism of action [72].

The substantial impact of HQ on both *A. fischeri* and *D. magna* suggests potentially significant effects on river ecosystems. However, assessing its effects on complete communities, such as microbial ones, is essential for a more realistic diagnosis.

**Figure 2:**
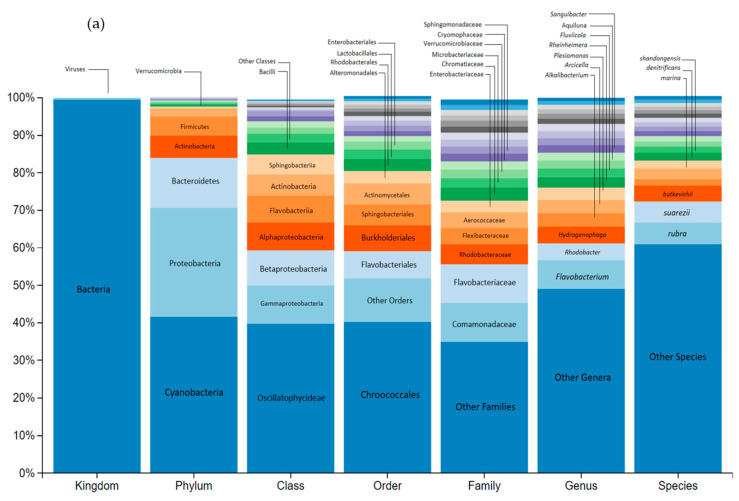

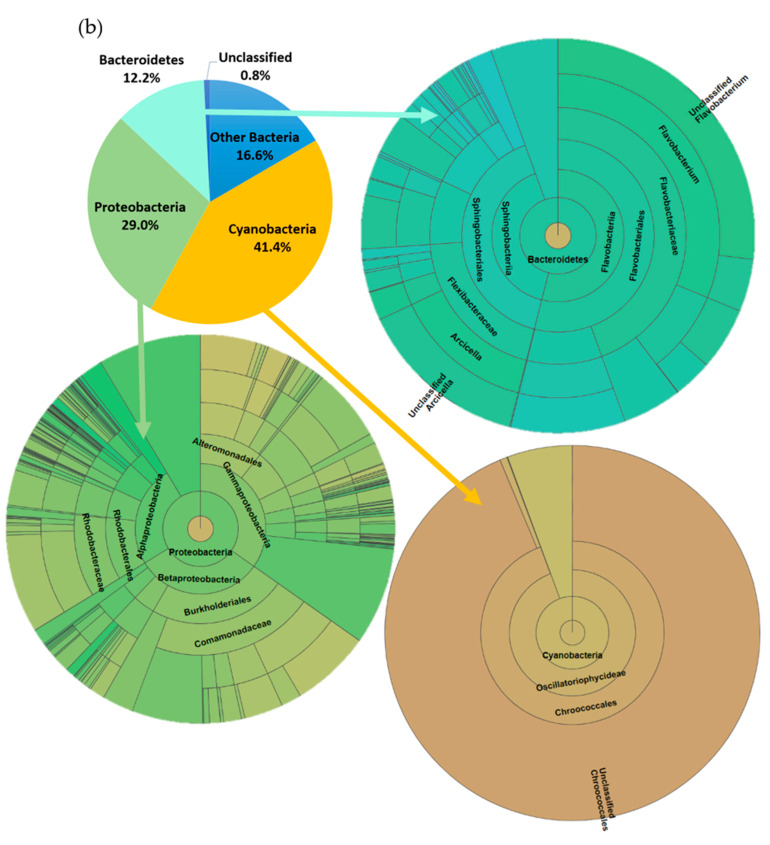
(**a**) Relative abundance of genetically sequenced microorganisms from a river, within their taxonomic classifications at each level. (**b**) Illustration of phyla that are most prominently observed in the river.

In Figure 2, the genetic sequencing of river microbial communities can be seen.

### 3.3. Impact on River Microbial Communities: Growth and Community-Level Physiological Profiling (CLPP) 

Figure 2 shows that river microorganism sequencing generated a total of 65,615 reads, all of which passed quality filters with a 100% success rate. Sequencing comprehensively covered all taxonomic levels, achieving >95% for phylum, class, and order, >50% for family and genus, and 23.33% for species. Figure 2a displays the most prevalent taxa (>2%) for river microorganisms at each taxonomic level. In Figure 2b, a visual representation is provided, illustrating the most prominently observed phyla with pie chart slices indicating their respective percentages.

The three predominant phyla were: *Cyanobacteria* (41.4% of the bacterial reads), *Proteobacteria* (29%), and *Bacteroidetes* (12.2%). Notably, 16.5% of bacterial reads remained unidentified, highlighting the presence of novel sequences in this study.

The *Cyanobacteria* phylogenetically belong to oxygenic phototrophic bacteria frequently found in rivers [74,75]. Almost all *Cyanobacteria* were classified within the class *Oscillatoriophycidaeae* (94.4%), with the majority falling under the order *Chroococcales*, a dominant group in freshwater biotopes [76].

Within *Proteobacteria*, we encountered three predominant classes: *Gammaproteobacteria* and *Betaproteobacteria*, exhibiting similar abundances at 34.7% and 31.5%, respectively, and *Bacteroidetes* at 13.22%. *Proteobacteria*, a prolific phylum of Gram-negative bacteria in freshwater bacterial communities [77] demonstrates rapid growth in response to organic nutrients [78]. *Gammaproteobacteria*, known for its high taxonomic diversity, featured *Alteromonadales* as the most prevalent order (31%), a representative of river microbial communities [79,80]. Notably, the order *Pseudomonadales* (8% of *Gammaproteobacteria* reads) includes the *Pseudomonadaceae* and *Moraxellaceae* families, some of whose members, such as *Pseudomonas*, play an active role in the degradation of phenolic compounds [81,82]. *Betaproteobacteria* were predominantly of the *Burkholderiales* order (74.7%), and among the *Alphaproteobacteria*, *Rhodobacterales* stood out (42.5%).

Within *Bacteroidetes*, Gram-negative anaerobic bacteria with significant involvement in the degradation of humic materials and polymers [78], we found two dominant classes: *Flavobacteria* (54% of *Bacteroidetes*) and *Sphingobacteriia* (40.5%).

Freshwater microbial communities have been suggested as excellent bioindicators for assessing the impact of micropollutants in river ecosystems [83] because disruptions at this level can have consequences throughout all trophic levels [83,84], leading to unpredictable effects on the ecological balance of the aquatic environment [85]. These communities serve as the foundation of the aquatic food web, particularly among primary producers, and also play a significant role in organic matter decomposition, thereby contributing to nutrient cycling and energy exchange, as well as the degradation of pollutants [86,87].

While our results indicate high toxicity of HQ in various aquatic indicators, it is surprising how the impact on the growth and metabolic capacity of these microbial communities appears to be buffered, as if these communities could effectively withstand HQ’s toxic effects.

**Figure 3:**
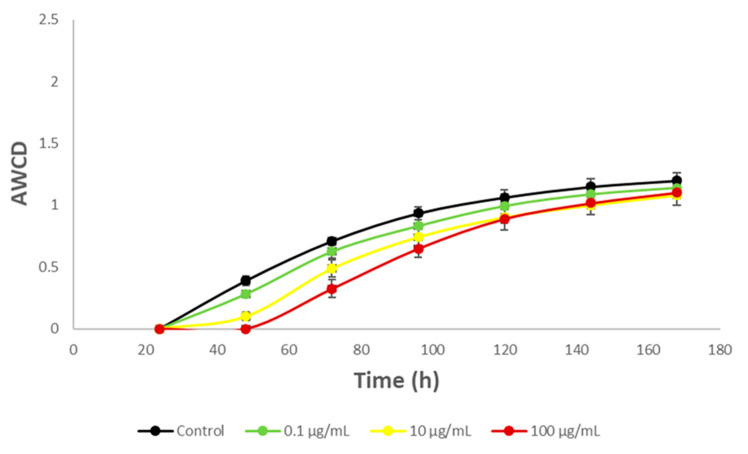
Average Well Color Development AWCD values of metabolized substrates in Biolog EcoPlates based on 168 h incubation of river microorganisms exposed to hydroquinone. Each point is the average value of three replicates.

**Figure 4:**
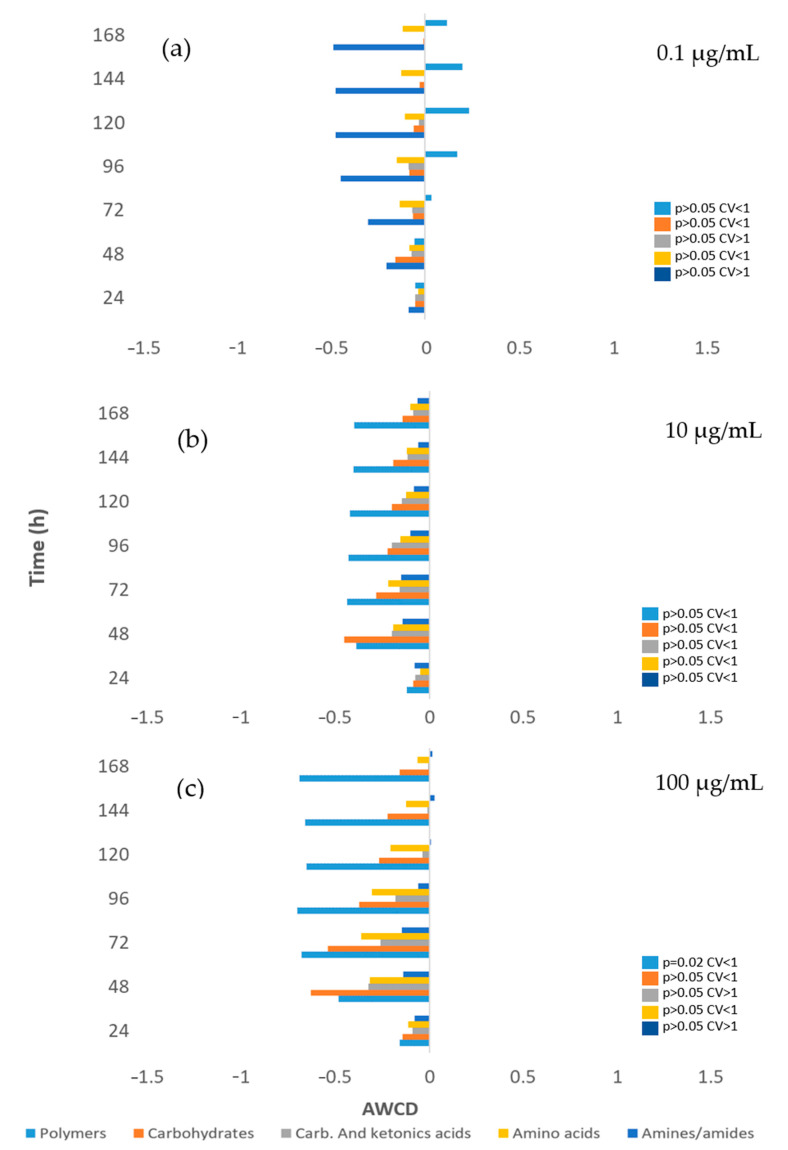
Metabolic effect differentiation by carbon sources of the river microorganisms exposed in different concentrations to hydroquinone with respect to the control (Y axis) ((**a**) 0.1 µg/mL; (**b**) 10 µg/mL; (**c**) 100 µg/mL). Each point is the average value of three replicates (Average Well Color Development-AWCD). The significance of differences from the control is indicated by *p*-values (t—Student), and the dispersion of values among the three replicates is represented by the coefficient of variation (CV).

In Figure 3 the effect of HQ on river microbial communities, measured as AWCD, can be seen. Furthermore, Figure 4 illustrates the impact of this product on the microbial profile of the community, compared to the control.

Furthermore, among the diversity of taxa, there may also exist varying metabolic capabilities, with certain bacteria potentially possessing mechanisms capable of degrading HQ. These microorganisms may derive greater advantages than others, potentially reshaping ecological interactions where the dominant flora that degrades HQ hydroquinone can be gradually formed [88,89]. As can be seen (Figure 3), although at the beginning there were small differences, after 72 h the growth of the community exposed to HQ practically matched those of the control, possibly due to these readjustments within the community.

Zhang et. al. [77] observed that concentrations of HQ at 100 mg/L (the highest tested in this study) in wastewater treatment plants resulted in the establishment of a stable community dominated by the same taxa we have identified in our samples (*Cyanobacteria*, *Proteobacteria*, and *Bacteroidetes*). These taxa showed minimal variation in their relative abundance compared to the control [32]. Specifically, the abundance of *Cyanobacteria* remained largely unaffected, *Bacteroidetes* showed a slight increase, and *Proteobacteria* exhibited a minor decrease in this study. The limited impact on Cyanobacteria, which constitute nearly half of our samples, may explain the minimal metabolic changes observed in our study, even at the highest concentration. Proteobacteria, as the largest group of Gram-negative bacteria with a wide range of metabolic pathways and a major role in the degradation of phenolic compounds [90], could withstand the HQ impact despite experiencing a modest decline (on the order of 10% at 100 mg/L) according to Zhang et. al. In fact, several members of this group present in our samples have been reported to be able to metabolize HQ.

Among the *Gammaproteobacteria* we found *Pseudomonadales* (specifically *Pseudomonas* genera) and members of the *Moraxellaceae* family, both proficient in utilizing and degrading HQ [80,81,91,92]. Additionally, within the Betaproteobacteria, we observed the presence of *Burkholderiales*, also capable of following HQ degradation pathways [7].

On the other hand, *Bacteroidetes* are known for their capacity to degrade various complex carbon compounds, including HQ [93], potentially increasing in number to compensate for the loss of Proteobacteria.

Beyond these changes in community structure reported, our results demonstrate that the final result of this taxonomic rearrangement within the community is that the metabolic capacity of the entire community is minimally affected by HQ (Figure 4). 

Only a decrease in the ability to metabolize polymers at the highest concentration of 100 µg/mL (*p* = 0.02) appears to occur. All other changes in the metabolic profile of the microbial community are not significant at any of the concentrations tested. This would be consistent with studies showing that functional genes for carbohydrate metabolism and energy metabolism were maintained at a high level following HQ exposure [32].

Therefore, although initially, the microbial flora was stressed by the influent HQ, which may even trigger the secretion of secondary metabolites that increase toxicity [32,33] the microbial community, after a succession of biological communities, gradually forms a dominant flora capable of degrading or tolerating HQ. As a result, the metabolic capacity of the microbial community remains stable, and it is foreseeable that the impact of HQ on rivers will be minimal.

In many countries, the implementation of maximum concentration limits for the industrial discharge of phenols has been established [5,94]. These limits typically range from low mg/L to μg/L, depending on the specific discharge location and the flow characteristics of the watercourse (EC, Commission Implementing Decision (EU), 2018) [95]. While these levels may provide protection for microbial communities, it is not necessarily guaranteed for other aquatic organisms, such as *D. magna.*

### 3.4. Impact of Hydroquinone on Allium cepa

**Figure 5:**
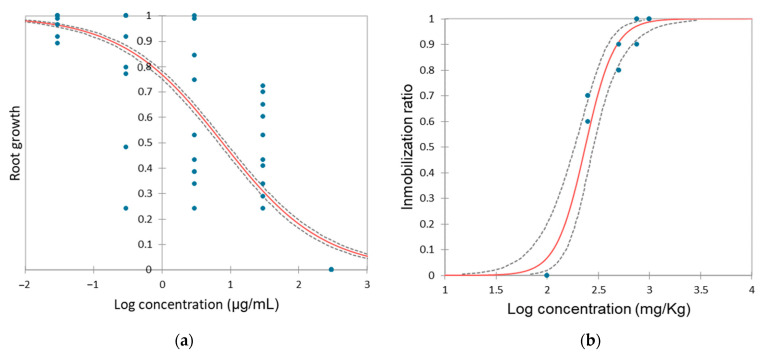
Dose–response curve for (**a**) *Allium cepa* and for (**b**) *Eisenia fetida* after 72 h and 14 days of exposure to hydroquinone, respectively. Red line represents the model and dashed lines indicate the confidence limits (95%). The points indicate the values of the replicates, in some cases the values may overlap.

HQ also exhibits phytotoxicity on *A. cepa*, significantly impacting bulb root growth. EC_50_ obtained was 7.631 (6.720–8.676) μg/mL and the dose–response curve after 72 h exposition is shown in Figure 5a.

While it has long been recognized that phenols can cause chromosomal fragmentation in *A. cepa* and disrupt root mitosis upon exposure [96], as far as our knowledge goes, the ecotoxicity of HQ on this plant has not been quantified before.

HQ demonstrates phytotoxicity on other plants as well: it reduces shoot growth in oats (*Avena sativa* L. ‘Goodfield’) and inhibits redroot pigweed [97], as well as impacting the growth of leaves, roots, and stems in common beans (*Phaseolus vulgaris*) [98]. Additionally, it exerts a phytotoxic effect on the germination of the plant species *Trigonella foenum-graecum* [99].

Previous reports have suggested changes in the polarization of the plant cell membrane after exposure to HQ, which could impact substance transport, although this effect appears to be minor in explaining cell death [98]. Probably, the primary mode of action of HQ involves significant damage to cellular membrane integrity, leading to a loss of metabolic activities and macromolecules, accompanied by associated oxidative stress [24]. Damaged cells may then initiate an apoptosis process [99].

### 3.5. Impact of Hydroquinone on Eisenia fetida

Our results demonstrate that, despite *E. fetida* being the most resilient bioindicator among the four tested, it still exhibits detectable toxicity. The dose–response of the earthworm exposed to HQ can be seen in Figure 5b with LC_50_ of 234.05 (184.13–281.18) mg/kg. When comparing the toxicity values of HQ on *E. fetida* to other phenolic compounds of plant origin (non-quinones), such as tannic acid, the latter shows much higher values (LC_50_ > 2000 µg/L) [100]. However, to the best of our knowledge, the impact of HQ on earthworms, particularly *E. fetida*, has not been previously investigated. While some evidence of toxicity can be found in the literature, it often pertains to compounds within the HQ family or chemically distinct derivatives, and it may involve different earthworm species. For instance, exposure studies involving various polyesters containing HQ, among other compounds, showed an *E. fetida* survival rate exceeding 80% after 14 days, suggesting a moderate level of toxicity to these bioindicators [101].

Interestingly, Osman [102] observed that additional earthworm species, including *L. rubellus* and *A. chlorotica*, seem to exhibit susceptibility to oxidative stress induced by quinones. This susceptibility may be attributed to their deficiency or notably low levels of DT-diaphorase, an enzyme recognized for its significant role in quinone detoxification.

The exposure of earthworms to HQ can occur through the ingestion of particles carrying the active product [103] and through percutaneous means. Earthworms possess a highly water-absorbent and water-loss-tolerant cuticle, allowing for significant water exchange through the body wall [104]. HQ’s relatively low molecular weight and slight hydrophobic nature could enable its permeability in biological membranes [105]. However, it is likely that ingestion, in this case, is what triggers the cytotoxic effects.

Earthworms play a crucial role in soil health and fertility as they decompose organic matter and mix the soil, improving its structure and enhancing its ability to retain water and nutrients, thereby allowing plants to access these nutrients. Therefore, their decline or reduction can have significant consequences for soil fertility [106].

The activity of these organisms is intimately connected to that of soil microorganisms, as earthworms have an important role in promoting microbial activity, likely by feeding on microorganisms or by selecting and stimulating specific microbial groups [107].

### 3.6. Impact on Soil Microbial Communities: Growth and Community-Level Physiological Profiling (CLPP) 

**Figure 6:**
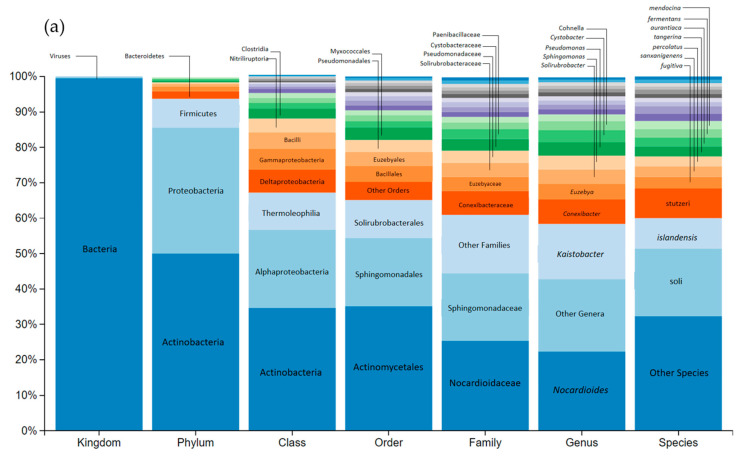

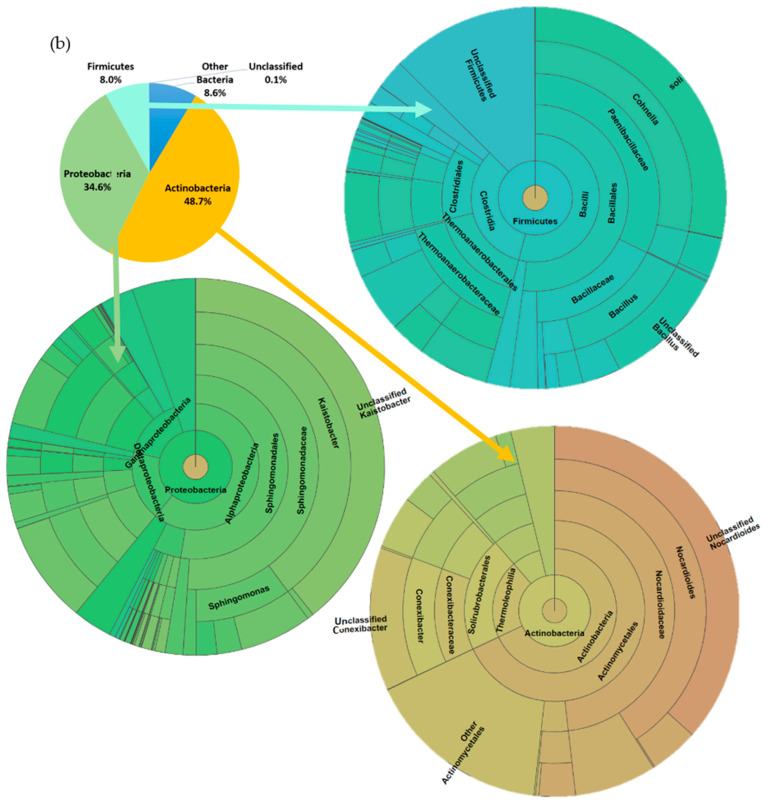
(**a**) Relative abundance of genetically sequenced microorganisms from a river within their taxonomic classifications at each level. (**b**) Illustration of phyla that are most prominently observed in soil. The significance of differences from the control is indicated by *p*-values (t—Student), and the dispersion of values among the three replicates is represented by the coefficient of variation (CV).

Figure 6 shows the great diversity of soil taxa. In this case the total reads were 61,347 and 100% passed quality filtering. It was possible to identify >90% of taxa at the taxonomic level of Phylum, Class, Order and Family, 88.63% at Genus and only 24.23% at species level. Figure 6a displays the relative abundance of the main taxons within each taxonomic level of the most prevalent taxa (>2%). Figure 6b, a visual representation highlights the most prominently detected phyla.

In our samples, we observed a predominance of two bacterial phyla: *Actinobacteria*, which constituted 48.7% of the bacterial reads, and *Proteobacteria,* making up 34.6% of the composition. Additionally, we detected a smaller proportion of *Firmicutes*, accounting for 8.0% of the total reads. This taxonomic distribution aligns with the typical bacterial diversity encountered in uncontaminated edaphic ecosystems where *Proteobacteria* are usually very abundant [108,109], *Actinobacteria* phyla are well represented [82] and *Firmicutes* are frequently detected [110,111,112].

Among the *Actinobacteria*, the Class *Actinobacteria* predominates (68.0%), practically all belonging to the order *Actynomycetales*, ubiquitous in different soil types [110,111,113,114]. More than half of *Proteobacteria* were *Alphaproteobacteria* (60.9%) followed by *Deltaproteobacteria* (17.9%) and *Gammaproteobacteria* (15.7%). Almost all *Alphaproteobacteria* are of the order *Sphingomonadales* with a small representation of the order *Rhizobiales* (8.11% of the *Alphaproteobacteria*). Among the *Deltaproteobacteria*, *Myxococcales* predominate (52.9%), all of them belonging to the family *Cystobacteraceae* and the genus *Cystobacter.* In *Gammaproteobacteria*, all the *Pseudomonadaceae* family are *Pseudomonas*. Among the *Firmicutes*, *Bacilli* (54.1%) and *Clostridia* (33.3%) are the predominant class.

**Figure 7:**
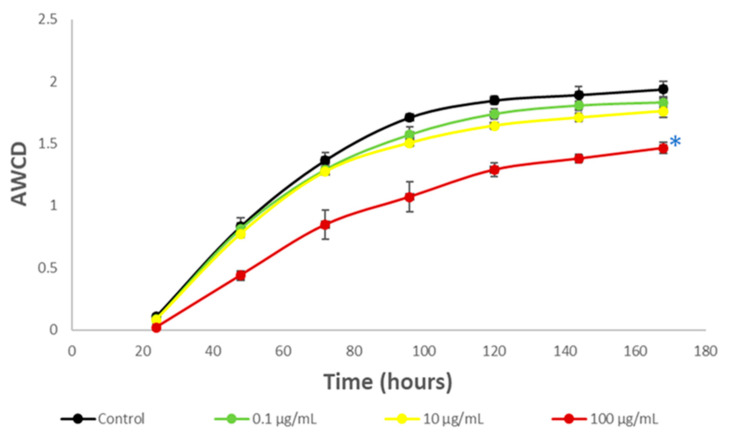
Average Well Color Development AWCD values of metabolized substrates in Biolog EcoPlates based on 168 h incubation of soil microorganisms exposed to hydroquinone. Each point is the average value of three replicates. Blue asterisks indicate *p* = 0.05.

In Figure 7, the effects of HQ on community growth measured as AWCD are depicted.

As can be observed, microbial communities also appear to withstand HQ exposure well, except at concentrations greater than 100 µg/mL (*p* = 0.05). In this case, there is no initial growth decline followed by subsequent recovery, as seen in the case of river microorganisms. Instead, at 100 µg/mL, growth is partially inhibited right from the beginning of HQ exposure. This heightened sensitivity of soil microbial communities compared to aquatic ones is consistent with findings from other studies where soil or sediment microorganisms seem to be more vulnerable to potentially toxic compounds than aquatic microorganisms [115,116]. This observation has also been noted for products or extracts of plant origin [51,117].

**Figure 8:**
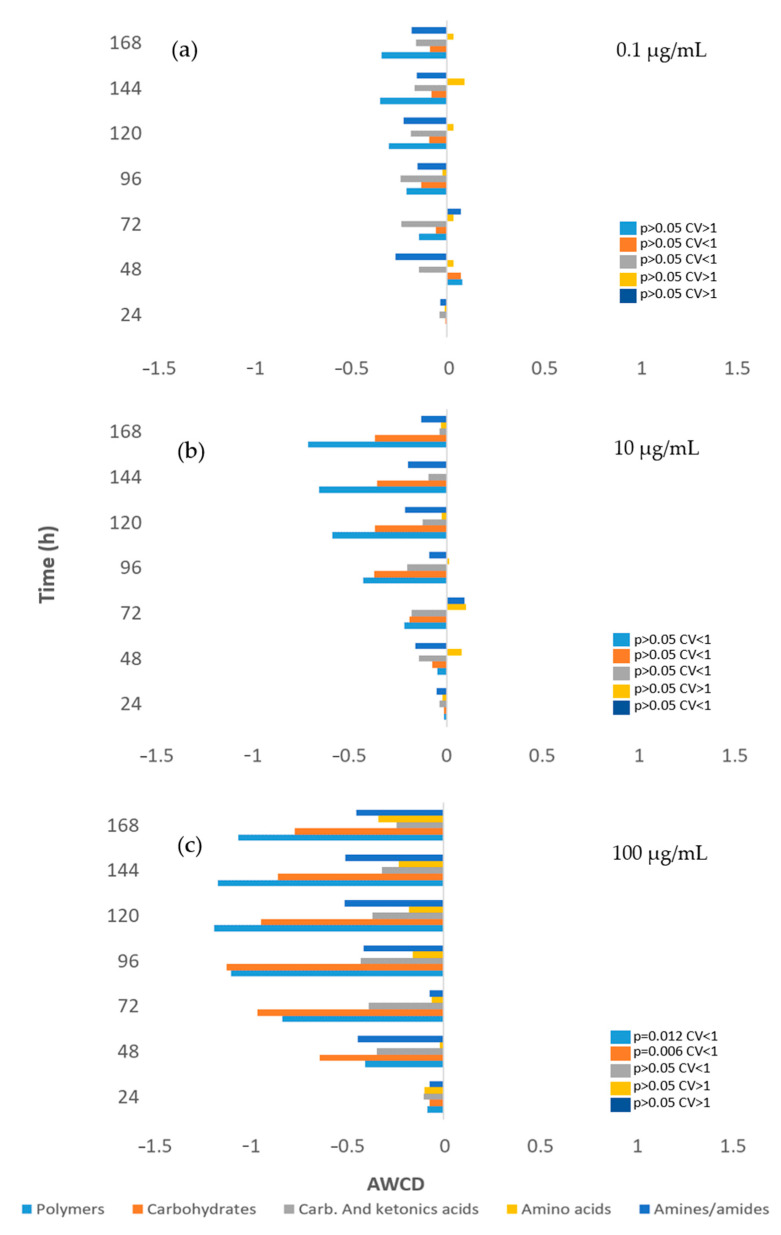
Metabolic effect differentiation by carbon sources of the soil microorganisms exposed in different concentrations to hydroquinone with respect to the control (O axis) ((**a**) 0.1 µg/mL; (**b**) 10 µg/mL; (**c**) 100 µg/mL). Each point is the average value of three replicates (Average Well Color Development (AWCD) in Time (h).

Moreover, at the metabolic level (see Figure 8), the concentration of 100 µg/mL induces a significant decrease in the ability to metabolize not only polymers (*p* = 0.012), as observed in the case of river microbial communities, but also carbohydrates (*p* = 0.006). Nevertheless, at lower concentrations, there are no significant changes in the metabolic profile (*p* > 0.05) for any metabolite.

There are very few studies that have examined the effect of HQ on soil microbial communities. Nevertheless, there is evidence that HQ may indeed impact microbial growth. Chen [118] observed that soils amended with HQ experience a decrease in the growth of cultivable microbial populations, with HQ being the most toxic dihydroxybenzene compared to other phenolic compounds such as resorcinol and catechol. It has also been reported that soil microorganisms’ exposure can lead to minor changes, such as an increase in the relative abundance of groups involved in fermentation and cellulolysis [36], which, in some way, may account for the slight variations in the metabolic profile we have detected.

The use of HQ as a urease inhibitor [21] to prevent urease from breaking down into urea, thus increasing the availability of NH_3_/NH_4_^+^ for plant uptake [119], has led to a limited number of studies examining the effect of HQ on soil microorganisms, especially in nitrification and denitrification processes, with varying results. On one hand, HQ, in line with our findings, appears to induce minimal changes in the community composition and functional profiles of the soil microbial community, with little impact on ureolysis groups [36,120]. However, other authors have reported that ammonia oxidation microbes were inhibited following HQ application [37] or that HQ delays urea hydrolysis, subsequently affecting nitrification and denitrification [121]. Nevertheless, there are limited reports on the effects of long-term HQ application on the soil nitrification and denitrification microbial community. Our results, however, do not indicate significant changes in the capacity to metabolize substrates potentially involved in nitrogen metabolism, such as carboxylic and ketonic acids, amino acids, or amines and amides.

The resilience exhibited by these soil microorganisms to HQ at concentrations below 100 µg/mL may stem from strategies akin to those described for aquatic microorganisms. In this scenario, we also encounter a significant diversity of taxonomic groups, making the replacement of sensitive species with more resistant ones, capable of degrading HQ, an expected occurrence. According to genetic sequencing, we have identified several genera, including *Pseudomonas* (3.31% of the total reads) and *Burkholderia* (Figure 6, within the section “other Proteobacteria”) and members belonging to the order *Rhizobiales*, all of them able to metabolize HQ [80,122,123]. Furthermore, as previously discussed, taxonomic groups within the *Sphingomonadaceae* family (constituting 19.6% of total soil reads) have been found to possess mechanisms for safeguarding against HQ exposure [124].

Other mechanisms, such as the production of specific enzymes for phenolic compound detoxification, as described in *Actinomycetales* members (constituting 32.94% of total reads in our samples) [125], and the formation of biofilms, as demonstrated by *Corynebacteriaceae* within the *Actinomycetales* order (representing 32.94% of total reads), able to metabolize HQ [126], are also plausible. In fact, the microbial diversity, structure, and function of a biofilm imparts a high metabolic capacity. It has been reported that biofilms are capable of removing more than 95% of phenolic compounds, including HQ [35].

Therefore, our findings suggest that unless occurring at exceptionally high concentrations rarely encountered in the environment, the impact of HQ on soil microbial communities is likely to have minimal effects on microbial growth and will not significantly impair their metabolic capacity.

## 4. Conclusions

This study demonstrates that HQ, a contaminant found in river ecosystems at concentrations on the order of µg/L, exhibits high toxicity to aquatic organisms such as *D. magna* and *A. fisheri*, as well as terrestrial indicators like the plant *A. cepa* and the invertebrate *E. fetida*. However, the concentration ranges at which ecotoxicity is observed (0.142–234 µg/mL) are several orders of magnitude higher than current environmental levels. Remarkably, both riverine and soil communities appear resilient to HQ exposure, exhibiting effects on growth or metabolic profiles only at the highest tested concentrations, notably 100 µg/mL. This resilience may be attributed to the diverse array of degradative and protective taxa within these microbial communities, which mitigate HQ impact in both aquatic and terrestrial environments.

Currently, entities such as the European Parliament and the Environmental Protection Agency (EPA) have instituted discharge limits for phenolic compounds at levels ranging from 0.05 µg/mL [127] to 1 µg/mL [128] orders of magnitude lower than those causing ecotoxicity in microbial communities but not necessarily in other aquatic organisms like *D. magna* or *A. fischeri*. It is important to consider that this exposure is persistent over extended periods and may interact with other toxins. Additionally, HQ frequently appears as an intermediate in the transformation of other compounds, potentially elevating its environmental levels. Cumulative effects, especially in soil, cannot be ruled out. Therefore, the toxicity values provided in this study should guide the maintenance and potential strengthening of discharge regulations, particularly to protect sensitive environments such as rivers and soils.

## Figures and Tables

**Table 1:** Physical and chemical properties of hydroquinone.

Hydroquinone Properties	
Molecular weight	110.11 g/mol [38]
Water solubility	73 g/L at 25 °C [38]
Melting point	170–172 °C [38]
Boiling point	287 °C [38]
Dipole moment	1.4–2.4 D [38]
Density	1341 kg/m [39]
Vapour pressure	2.34 × 10^−3^ Pa at 25 °C [39]
pH stability	4.0–7.0 [39]
Partition coefficient (log p_ow_)	0.59 [40]
pK_a_	pK_1_ = 9.9 pK_2_ = 11.6 [40]

## Data Availability

The original contributions presented in the study are included in the article, further inquiries can be directed to the corresponding author/s.

## Appendix A

**Table A1:** Analysis of the river water from which the microorganism samples were obtained.

**Physical-Chemical Analysis of the River Water Sample**	
HCO^−^_3_ (mg/L)	313
TDS (mg/L)	1925
MES (mg/L)	6
Cl^−^ (mg/L)	618 ± 93
SO_4_^2−^ (mg/L)	415 ± 62
NO_3_^−^ (mg/L)	17.7 ± 2.7
NO_2_^−^ (mg/L)	<0.05
F^−^ (mg/L)	0.071 ± 0.011
PO4^3−^ (mg/L)	0.6 ± 0.09
NH_4_^+^(mg/L)	<0.1
O_2_ (mg/L)	2.3
DQO (O_2_) (mg/L)	<25
DBO5 (mg/L)	<5
Ca (mg/L)	235 ± 80
Mg (mg/L)	38.1 ± 13.7
Na (mg/L)	415 ± 95
K (mg/L)	6.08 ± 1.95

**Table A2:** Analysis of the soil from which the microorganism samples were collected.

**Soil Composition**	**Surface Soil**	**30 cm Deep Soil**
Clay content (%)	20.98	23.61
Sand content (%)	16.08	13.10
Silt content (%)	62.94	63.29
pH	7.9 ± 0.5	8.1 ± 0.5
K (mg/L)	238 ± 40	208 ± 35
Mg (mg/Kg)	244 ± 39	242 ± 39
P Oslen (mg/Kg)	13 ± 2	10 ± 1.7
EC_1:5_ (dS/m)	0.6 ± 0.09	0.4 ± 0.06
Organic matter (g/100 g)	2.46 ± 0.31	2.35 ± 0.30
